# Patient advocacy organizations’ information for patients on pre-approval access to investigational treatments

**DOI:** 10.1186/s13104-019-4745-7

**Published:** 2019-10-28

**Authors:** Kelly McBride Folkers, Sarah Leone, Arthur Caplan

**Affiliations:** Division of Medical Ethics, Department of Population Health, NYU School of Medicine, Translational Research Building, 227 E. 30th Street #754B, New York, NY 10016 USA

**Keywords:** Clinical trials, Expanded access, Pre-approval access, Patient advocacy organizations

## Abstract

**Objective:**

To evaluate the availability of information regarding patient access to investigational treatments through clinical trials and non-trial pre-approval access pathways from a sample of patient advocacy organization (PAO) websites in the United States.

**Results:**

We systematically analyzed the content of 118 randomly selected PAO websites to assess whether they contained information on clinical trials and non-trial pathways—e.g., the U.S. Food and Drug Administration (FDA) expanded access (EA) program and right to try—over the course of two months from February to March 2019. A majority (81%, n = 96) of PAOs provided a link to ClinicalTrials.gov, and 73% (n = 86) had their own clinical trial finder or list of relevant trials. 23% (n = 27) mentioned EA, with 8% (n = 9) providing specific resources for FDA’s EA program. 8% (n = 10) provided a statement on the passage of the federal right to try law. A majority of PAO websites contained information on clinical trials, but a minority discussed non-trial pre-approval access. The lack of information on the latter highlights an area in need of improvement.

## Introduction

In the Internet era, patients play an ever-growing role in their own care. Patients with serious, rare, or life-threatening conditions often turn to various Internet sources to research potential treatments. Patients may seek information from patient advocacy organizations (PAOs), or communities of individuals with a particular health condition in search of a treatment or cure. PAOs, in conjunction with government organizations, industry, policymakers, and community-based organizations, play a key role in educating the patients and the public about new treatments in development for particular health conditions [1].

In recent decades, increasing numbers of patients have sought access to investigational medical products for a variety of reasons. For some with rare diseases, no standard of care exists, and thus, the only option for curative therapy is an investigational agent. In other scenarios, patients may have exhausted approved treatment options and seek opportunities to try investigational products. Patients gain access to investigational drugs most commonly by participating in clinical trials [2]. Patients who do not qualify for clinical trials can obtain these drugs through alternative regulatory pathways. The U.S. Food and Drug Administration (FDA) allows patients with terminal illness to request access to investigational drugs through its expanded access (EA) program [2]. Patients, through their physicians, can request investigational products from pharmaceutical and academic sponsors. The FDA and an institutional review board (IRB) review these requests before the patient can be treated. The FDA allows over 99% of these requests to proceed [3]. The EA program, however, has not satisfied all stakeholders, as some perceive the FDA’s regulatory purview to be a barrier for efficient and timely access to investigational medical products [4]. In May 2018, a federal right to try (RTT) law was enacted, which created a new pathway that does not require FDA review or IRB approval [5]. As of this writing, we are aware of two patients have received access through RTT [6, 7]. Because the law has received significant media attention, some have predicted that increased demand for investigational drugs is likely [4].

Patients may learn about investigational medical products in several ways. First, they may search for clinical trials recruiting patients on ClinicalTrials.gov, a resource provided by the U.S. National Library of Medicine that lists publicly and privately funded clinical trials globally [8]. The site may not have reliable information for patients in all instances; many sponsors have failed to comply with mandatory results reporting on the site [9]. Second, they may use online navigators for EA from private foundations [10, 11]. Third, they may find information about how to access investigational medical products on pharmaceutical company websites. Fourth, they may speak to their physicians about these opportunities, but some physicians are not equipped to provide investigational medical products or are not aware of non-trial pathways. Finally, patients may turn to PAOs, particularly if they use Google or other search engines or social media to research their conditions and available treatment options/investigational products.

If patients and their physicians are not aware of the various regulatory pathways that allow them to access investigational medical products, they may not be able to make fully informed choices regarding their care [12–14]. As patients and families may turn to Internet resources to research possible treatment options, it is important to understand the breadth of information available online. The prevalence of publicly available expanded access policies on pharmaceutical companies websites has been previously analyzed [15], but the information on PAO websites has not yet received the same scrutiny. We analyzed a sample of PAO websites to evaluate the availability of information regarding access to investigational treatments.

## Main text

### Methods

There is no comprehensive database of PAOs operating in the United States. We randomly selected 134 disease-specific PAOs that are members of the National Health Council (NHC), National Organization for Rare Disorders (NORD), and/or have been invited to participate in educational webinars hosted by Janssen Pharmaceuticals. NHC and NORD list member organizations on their webpages; the Janssen list was generously provided by Patient Support in the Office of the Chief Medical Officer at Janssen.

We confirmed that these PAOs are currently operating and classified as public charity 501(c)(3) organizations using GuideStar. PAOs not listed on GuideStar or were determined to have a primary focus of political advocacy/lobbying were excluded. Our final sample was 118 PAOs.

We analyzed each website’s content on clinical trial information, expanded access, and right to try using specific metrics over the course of two months from February to March 2019 by using the same set of search terms in each website’s native search bar (i.e., “compassionate use,” “expanded access,” “clinical trials,” and “right to try.”). If a search did not return results or the site lacked a search bar, we manually navigated through the site’s pages until we found the appropriate information. If we were unable to find relevant information, we scored the site as lacking the information. To identify relevant information on PAO websites, we asked the following questions: (1) Did the website include a link to ClinicalTrials.gov? (2) Did the website include a native clinical trial finder or list or clinical trials? (3) Did the website include any information on expanded access/compassionate use? (4) Did the website include information on right to try laws? (5) Did the information include a list of investigational drugs for the health condition of interest? (6) Did the website include information or a link to FDA resources on expanded access? One researcher scored each category or question with a metric of “yes” or “no” and reviewed the data a second time for quality control. Discrepancies or areas of confusion, which were encountered in fewer than 15% of websites, were noted and reviewed by two researchers who reached a consensus.

### Results

**Fig. 1:**
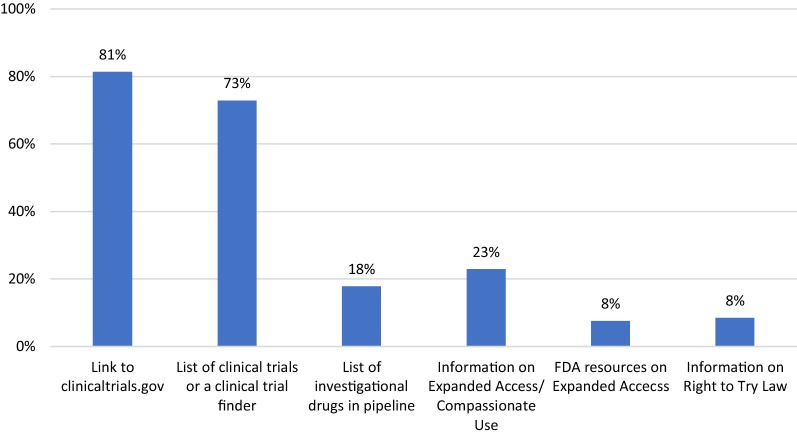
Analysis of patient advocacy organization (PAOs) and the extent to which they provide information and resources on clinical trials, the U.S. Food and Drug Administration’s expanded access program, and the Right to Try Act. Bars represent the percentage of PAOs that provided information on 7 different criteria presented in the “Methods” section

A majority (81%, n = 96) of PAOs provided a link to ClinicalTrials.gov, and 73% (n = 86) had their own clinical trial finder or list of relevant trials (Fig. 1). 23% (n = 27) mentioned EA, with 8% (n = 9) providing specific resources for the FDA’s EA program, such as a link to the FDA’s page on EA or instructions for how to request EA for an investigational product. 8% (n = 10) provided a statement on the passage of the federal RTT law.

Though no information was presented inaccurately, navigational functionality of each site and the amount and presentation of information on each topic, if present, varied greatly. For example, some sites contained complete information on the FDA’s expanded access program, while others mentioned expanded access in blog posts, webinars, or in other materials not explicitly devoted to the subject.

### Discussion

PAOs can allow patients to identify opportunities for research participation and non-trial access to investigational drugs. While a majority of PAOs provided sufficient resources regarding clinical trial participation, as a whole they provided limited information regarding non-trial access. The majority of patients who receive access to investigational medical products do so through clinical trial participation, not EA, so it was expected that there would be more robust information across PAO websites on clinical trials. However, significant numbers of patients access investigational medical products outside of clinical trials each year, making non-trial access an option for many. While the exact number of patients who receive investigational medical products through EA each year is not known, it is estimated that thousands of patients participate in large cohort EA programs or use investigational products through individual requests [2, 3].

Not all patients qualify for clinical trials because they do not meet inclusion criteria; they have a co-morbid condition; or they live too far away from a clinical trial site for participation to be feasible. Patients who do not qualify for a clinical trial, or who cannot afford to travel to a clinical trial site or have other logistical barriers preventing them from participating may not be aware that they can access investigational products outside of a clinical trial. Previous scholarship on non-trial preapproval access has identified a lack of patient and physician education and knowledge of EA as a barrier for those seeking access to investigational medical products [16]. Thus, PAOs ought to serve the needs of those within their patient communities that require non-trial pre-approval access pathways by making them aware of these options. Though successfully obtaining non-trial pre-approval access to an investigational medical product does not guarantee that a patient’s condition will improve, these pathways may be the only options left for a subset of patients that have exhausted or lack other treatment options.

The passage of the Right to Try Act of 2017 has complicated matters regarding non-trial access, as the law created a new pathway for access that has thus far received little usage. On May 21, 2018, 104 PAOs sent a letter to Congress stating, “We write to express our strong opposition to the [Right to Try Act] [...] We reiterate our concern with creating a secondary pathway for accessing investigational therapies outside of clinical trials” [17]. The letter argues that RTT will not achieve its stated goal of increasing patient access to investigational medical products as it does not address true barriers to access like pharmaceutical company denials of EA requests. Of the 104 PAOs that signed this letter, 20 are included in this study, but only 10 mentioned RTT on their websites. Four organizations in our study that signed the letter expressed opposition to RTT on their websites. It is possible that the majority of PAOs have not advertised the RTT pathway because of low uptake.

### Conclusion

Autonomy is a fundamental principle of biomedical ethics. In many cases, patients can only make autonomous decisions when they have full access to all available options for their care. Physicians have an obligation to make patients aware of these options, but they may not be educated on the available regulatory pathways or equipped to provide investigational medical products. As such, the quality and quantity of resources PAOs provide could influence patient decision making. The lack of information regarding non-trial pre-approval access highlights areas in need of improvement.

## Limitations

The total number of PAOs operating in the United States is unknown, so our sample may not be representative. Navigational functionality of individual websites varies; relevant information may have been overlooked in some cases. PAOs vary in size and revenue, and they may have industry partnerships that preclude the inclusion of any information regarding investigational treatments that could be construed as promotional. Gaps in the availability of information are not necessarily due to a lack of interest or motivation to provide resources to patients.

## Data Availability

The datasets used and/or analyzed during the current study are available from the corresponding author on reasonable request.

## Abbreviations

PAO: patient advocacy organization
EA: expanded access
FDA: Food and Drug Administration
IRB: institutional review board
RTT: right to try
NHC: National Health Council
NORD: National Organization for Rare Disorders
